# The General Practitioner and Research

**Published:** 1908-03-28

**Authors:** 


### The General Practitioner and Research.

In a recently published account of a visit to some German clinics it is mentioned that, amongst physicians there, the best known English name is that of Mackenzie of Burnley, which place we all know to be a North-country town unprovided with a medical school. It is not hard to appraise correctly foreign work in medical science, so that this must be reckoned high praise, and an encouragement to general practitioners with aspirations to research. The quarter from which it comes, too, is an authoritative one. The reason why much more of original work, both valuable and otherwise, is done in Germany than in England is to be found partly in the divergent systems of professional advancement current respectively in these two countries. There a reputation is built up largely by means of laborious original contributions to the study of a subject not necessarily utilitarian. Here, on the contrary, conspicuous success comes for the most part from a certain abiding impression of clinical ability made on fellow-workers by the successful candidate for a junior hospital appointment, which requires only academic triumphs, and carries with it the certainty of continuous promotion.

The way in which a general practitioner may do useful original work is probably somewhat as follows. In the first place he must be of an inductive turn of mind. Darwin said that the man who was not an active theoriser would make an indifferent observer. The two faculties, indeed, are bound up together. Regularities of Nature are perceived by those only who can fit theory after theory to their observations till they find one suitable; and to whom their scientific imagination renders facts interesting (as possible objects of correlation), and therefore salient. The aspirant must have the rare gift of a natural tendency to notice things he has not been taught to notice, and that others take no count of, and to jump to conclusions about them which are generally the right ones. In these prosaic days no cap, no hood, will carry such a gift with it. The point is important because a general practitioner's subject of investigation has to be one in which hypothesis counts for more, and verification for less, than usual. He has a wide field of observation, but only scanty equipment. Highly trained workers like the consultant and the specialist have at hand resources such as special libraries, or laboratories where assistants in search of subjects for theses will give devoted service. The practitioner must remember that a high standard of work is the only permissible one. In strong contrast to the pleasurably exciting mental flash of intuition is the lengthy and laborious process of testing and of proof, the exacting nature of which is summed up in the Platonic maxim that the doer must be at the beck of the thing to be done. Much of what is now dignified by the high name of research Sir Clifford Allbutt lately characterised as merely clerk's work; indeed, for labours like the above, as for the preliminary conception, few anywhere are competent. The lack of doctorates or demonstratorships, however, need not discourage. "They do most by books," says Sir Thomas Browne, "who could do much without them; and he that chiefly owes himself unto himself is the substantial man."

